# Expression of the fusogenic p14 FAST protein from a replication-defective adenovirus vector does not provide a therapeutic benefit in an immunocompetent mouse model of cancer

**DOI:** 10.1038/cgt.2016.41

**Published:** 2016-10-14

**Authors:** C M Wong, L A Nash, J Del Papa, K L Poulin, T Falls, J C Bell, R J Parks

**Affiliations:** 1 Regenerative Medicine Program, Ottawa Hospital Research Institute, Ottawa, Ontario, Canada; 2 Department of Biochemistry, Microbiology and Immunology, University of Ottawa, Ottawa, Ontario, Canada; 3 Centre for Neuromuscular Disease, University of Ottawa, Ottawa, Ontario, Canada; 4 Cancer Therapeutics Program, Ottawa Hospital Research Institute, Ottawa, Ontario, Canada; 5 Department of Medicine, University of Ottawa, Ottawa, Ontario, Canada

## Abstract

When injected directly into a tumor mass, adenovirus (Ad) vectors only transduce cells immediately along the injection tract. Expression of fusogenic proteins from the Ad vector can lead to syncytium formation, which efficiently spreads the therapeutic effect. Fusogenic proteins can also cause cancer cell death directly, and enhance the release of exosome-like particles containing tumor-associated antigens, which boosts the anti-tumor immune response. In this study, we have examined whether delivery of an early region 1 (E1)-deleted, replication-defective Ad vector encoding the reptilian reovirus p14 fusion-associated small transmembrane (FAST) protein can provide therapeutic efficacy in an immunocompetent mouse tumor model. A high multiplicity of infection of AdFAST is required to induce cell fusion in mouse mammary carcinoma 4T1 cells *in vitro*, and FAST protein expression caused a modest reduction in cell membrane integrity and metabolic activity compared with cells infected with a control vector. Cells expressing FAST protein released significantly higher quantities of exosomes. In immunocompetent Balb/C mice harboring subcutaneous 4T1 tumors, AdFAST did not induce detectable cancer cell fusion, promote tumor regression or prolong mouse survival compared with untreated mice. This study suggests that in the context of the 4T1 model, Ad-mediated FAST protein expression did not elicit a therapeutic effect.

## Introduction

Finding new and effective therapeutics is an ongoing challenge in the cancer field. Over the years, many pre-clinical research efforts and clinical trials have utilized viruses in an attempt to combat the disease. Indeed, an oncolytic herpes simplex virus expressing granulocyte macrophage colony-stimulating factor has recently been approved for clinical use in North America.^1^ For one of the most commonly used gene therapy vectors, adenovirus (Ad), two Ad-based vectors have been approved for cancer treatment in China—a non-replicating Ad encoding p53, termed Gendicine, and an oncolytic Ad deficient in the E1B-55 kD protein.^2^ However, a common problem with Ad is their restricted ability to disperse throughout a tumor, particularly following direct intratumoral injection.^3, 4, 5^ Typically, Ad infection is limited to a 5 mm area surrounding the injection tract, which prevents efficient transgene product delivery to all cells of the tumor.^4^ Although this may not be a significant issue when Ad is used to delivery the gene for secreted proteins, such as cytokines, it may limit the efficacy of other types of therapeutic genes and proteins, which require expression directly within the cell to exert their effect, such as, for example, the tumor supressor p53.

To increase the proportion of the tumor impacted by the Ad vector, several research groups have encoded genes for fusogenic proteins within the vector. Infection of a single cell would result in expression of the fusogenic protein and any other encoded, therapeutic protein, and subsequent cell–cell fusion would amplify the therapeutic effect to a greater percentage of the tumor mass. Various viruses have been modified to express membrane fusion proteins from Gibbon ape leukemia virus (GALV), respiratory syncytial virus (RSV), measles virus (MV), Newcastle disease virus and human immunodeficiency virus type 1 (HIV-1), and in many studies these vectors were shown to be effective in tumor regression.^6, 7, 8, 9, 10, 11, 12, 13, 14^ Ad vectors expressing GALV, RSV, MV or vesicular stomatitis virus (VSV) membrane fusion proteins as a sole anti-cancer agent were able to reduce tumor burden in xenograft and immunocompetent mouse models in the absence of other complementing anti-cancer therapeutic transgenes.^8, 9, 12, 13^ Thus, in many studies, the process of cancer cell fusion, and associated disruption in cellular processes, can lead to tumor regression.

Several studies have shown that enhanced immune activation also contributes to the anti-cancer efficacy of fusogenic proteins. Cell fusion can activate a subset of interferon stimulated genes (but not interferon itself) through an interferon regulatory factor 3 (IRF3)-dependent pathway and does not require external interferon signaling.^15^ Expression of fusogenic proteins in cancer cells also causes the release of exosome-like molecules called syncytiosomes. These syncytiosomes contain tumor antigens and are effective in priming dendritic cells (DC) to promote an anti-cancer immune response.^16^ Dying syncitia shed higher levels of these syncytiosomes that can promote macrophage activation and DC cross presentation of tumor-associated antigens, resulting in cancer-specific T-cell production and a long lasting anti-tumor immunity.^17, 18^

The reptilian reovirus p14 fusion-associated small transmembrane (FAST) protein is a 125 amino acid type III transmembrane protein.^19, 20^ The small size of p14 FAST relative to other fusogenic proteins is particularly attractive as it would leave sufficient cloning room within the Ad vector to accommodate additional therapeutically-relevant genes. Several studies have shown that expression of the FAST protein in cancer cells in culture can result in wide-spread syncytia formation, leading to apoptosis/cell death.^21, 22, 23^ Mice injected with VSV-expressing p14 FAST protein had higher viral titers from infected tissue *in vivo*, suggesting a greater ability to spread and replicate compared with the control virus.^24^ Co-administration of an oncolytic VSV armed with the p14 FAST protein increased oncolytic vaccinia virus titers and synergistically promoted cancer cell death *in vitro.*^25^

In previous work, we examined whether Ad-mediated delivery of FAST to cancer cells could lead to cell death in the human adenocarcinoma A549 cell line *in vitro* and lead to tumor regression and increased survival in an A549 xenograft tumor model in immunodeficient mice *in vivo.*^23^ We showed that expression of FAST protein from an E1-deleted Ad vector caused extensive cell fusion of A549 cells in culture, resulting in loss of cellular metabolic activity and membrane integrity, which correlated with induction of apoptosis. Unfortunately, in the A549 xenograft CD-1 nude mouse cancer model, Ad-mediated FAST gene delivery did not induce detectable cell fusion, reduce tumor burden nor enhance mouse survival compared with the controls. However, any contribution of FAST protein-mediated activation of anti-tumor immunity could not occur in the A549 xenograft mouse model that used immunodeficient mice. Thus, we have now evaluated AdFAST efficacy in an immunocompetent mouse of cancer.

## Materials and methods

### Cell culture

Mouse colon carcinoma CT26, melanoma B16F10 and mammary carcinoma 4T1 cells were maintained in Dulbecco's Modified Eagle Medium (DMEM) (Sigma Aldrich, Oakville, ON, Canada) supplemented with 10% (v/v) fetal bovine serum (FBS) (Sigma Aldrich), 2 mm GlutaMAX (Invitrogen, Waltham, MA, USA) and 1 × antibiotic-antimycotic (Invitrogen). Human lung adenocarcinoma A549 cells were grown in Modified Eagle Medium (MEM) with similar supplements. Cells infected with Ad were maintained in medium as described above, except with 5% FBS instead of 10% FBS. All cells were kept at 37 °C and in a 5% CO_2_ atmosphere.

### Adenoviral constructs and adenovirus purification

All vectors used in the study are based on early region 1 (E1)- and E3-deleted Ad serotype 5, and have been described previously.^23, 26, 27, 28^ AdEmpty does not encode a transgene.^26^ AdRFP expresses the monomeric red fluorescent protein (a kind gift of Roger Tsien, University of California, San Diego^29^) under the control of the human cytomegalovirus (CMV) enhancer/promoter and bovine growth hormone polyadenylation sequence (BGHpA).^27^ AdmCherry is similar in structure to AdRFP, but expresses the mCherry mutant of the *Discosoma* sp. red fluorescent protein.^30^ AdFAST contains the p14 FAST open reading frame under regulation by the CMV enhancer/promoter and BGHpA.^23^ AdFAST-HA has been previously described,^23^ and is similar in structure to AdFAST, but contains an HA epitope tag on the C terminus of the FAST protein. AdFAST/RFP expresses the FAST protein and RFP under separate CMV promoters and BGHpA.^23^ Adβ-Gal has the *E. coli* β-galactosidase gene under control by the CMV enhancer/promoter.^28^ Adenoviral stocks were amplified and purified as described previously.^31^

### Fluorescence microscopy

CT26, B16F10 and 4T1 cells were seeded at a density of 0.8x10^6^ cells per 35 mm plate. Next day, the cells were infected at an multiplicity of infection (MOI) of 50 with AdRFP or AdFAST/RFP for 1 h in 200 μl inoculum. Two ml of media was added and cells were viewed using the Zeiss Axiovert 200 M microscope (North York, ON, Canada) (20 × objective) every 24 h. Images were compiled in Adobe Photostop CS4.

### Membrane integrity assays

CT26, B16F10 and 4T1 cells were seeded at 2 × 10^4^ cells per well in 96 well plates and grown to sub-confluency. Cells were infected at varying MOI with AdEmpty or AdFAST in a 25 μl volume for 1 h, followed by addition of 100 μl of 5% FBS/DMEM. For all experiments, the VSVΔ51 virus^32^ and cells lysed using 9% Triton X-100 for 45 min before starting the assay were used as positive controls. Each treatment condition was conducted in triplicate. Seventy-two hours post infection, 50 μl from each well was transferred to a new 96 well plate. To examine membrane permeability, the CytoTOX-ONE Homogeneous Membrane Integrity Assay kit (Promega, Madison, WI, USA) was used according to the manufacturer's instructions. Fluorescence readings were obtained using a 544 nm excitation and 590 nm emission filter on the FluoSTAR Galaxy fluorimeter (BMG Labtech, Guelph, ON, Canada).

### Ad infectivity and promoter activity

4T1 and A549 cells were plated at a density of 0.8 × 10^6^ cells per 35 mm dish. When plates were confluent, cells were infected with Adβ-Gal at an MOI of 1. Twenty-four hours post infection, cells were either fixed and stained with 50 μl ml^−1^ X-gal overnight^33^ or collected to determine β-galactosidase activity using the Galacto-Star β-Galactosidase Reporter Gene Assay System for Mammalian Cells (Applied Biosystems, Thermofisher, Waltham, MA, USA). Chemiluminescent activity was determined using a 20/20n luminometer (Turner Biosystems, Madison, WI, USA).

### Immunoblot analysis

4T1 cells were seeded at 1x10^6^ cells per 35 mm dish. Next day, the cells were infected for 1 h at the indicated MOI of Ad virus. Following a 48 or 72 h incubation, whole-cell lysates were collected using 2 × SDS/PAGE protein loading buffer (62.5 mm Tris HCl pH 6.8, 25% glycerol, 2% SDS, 0.01% bromophenol blue, 5% β-mercaptoethanol). Samples were boiled for 5 min, separated by electrophoresis on a 15% SDS-polyacrylamide gel, and transferred onto a polyvinylidene fluoride (PVDF) membrane (Millipore, Etobicoke, ON, Canada). The resulting membrane was probed with a mouse HA tag monoclonal antibody (1:10 000, Cell Signaling (Beverly, MA, USA) #2367), rabbit cleaved caspase-3 monoclonal antibody (1:1000, Cell Signaling #9664) or full-length caspase-3 antibody (1:1000, Cell Signaling #9662) and 1:5000 goat anti-rabbit IgG (Bio-Rad, Mississauga, ON, Canada, #170-6515) or goat anti-mouse IgG antibody conjugated to horseradish peroxidase (HRP) (1:10 000, Bio-Rad #170-6516). The membrane was also probed with antibody to α-tubulin to confirm equal loading (1:5000 rabbit α-tubulin antibody, AbCam (Toronto, ON, Canada) #ab15246, 1:5000 goat anti-rabbit IgG conjugated to HRP, Bio-Rad #170-6515). Blots were developed using the Pierce Enhanced Chemilumescent (ECL) Western Blotting Substrate (Thermo Scientific, Waltham, MA, USA).

### Giemsa staining

4T1 cells were seeded in 4 well plates (0.15 × 10^6^ per well) and grown to confluence. Cells were infected with AdEmpty or AdFAST at an MOI of 1000 for 48 h. Cells were fixed and stained with Giemsa stain (Merck Millipore, Etobicoke, ON, Canada) and visualized using the Zeiss Axiovert 200 M microscope (20 × objective).

### MTS metabolic activity assays

4T1 cells were plated in 96 well plates at a density of 2x10^4^ cells per well. Next day, wells were infected with AdEmpty, AdmCherry, or AdFAST for 1 h in 25 μl. Following the 1 h infection, 5% FBS/MEM was added to a final volume of 100 μl. The relative metabolic activity was determined over a time course of 96 h, with 24 h intervals, using the CellTiter 96 AQueous Non-Radioactive Cell Proliferation Assay (Promega). For the assay, cells were incubated for 1 h with MTS substrate (3-(4,5-dimethylthiazol-2-yl)-5-(3-carboxymethoxyphenyl)-2-(4-sulfophenyl)-2H-tetrazolium). Absorbance readings were obtained at 490 nm using the SpectraMax 190 plate spectrophotometer (Molecular Devices, Sunnyvale, CA, USA).

### Exosome isolation

4T1 cells were plated in 60 mm plates at a density of 1.6 × 10^6^ cells per well. Next day, cells were infected with AdEmpty or AdFAST at an MOI of 1000 for 1 h in 500 μl. Following the 1 h infection, 5% FBS/MEM was added to a final volume of 3 ml. Media was isolated 72 h post infection, and exosomes were isolated using the Exoquick kit (System Biosciences, Mountain View, CA, USA) according to the manufacturer's instructions. Purified exosomes were analyzes by immunoblot using rabbit anti-Alix (Sigma, Oakville, Ontario, Canada) at a dilution of 1:1000, rabbit anti-Flotillin-2 (Cell Signaling, Danvers, MA, USA) at 1:1000, or HA at 1:1000. The concentration of exosome particles isolated from media was quantified using Zetaview (Particlemetrix, Germany) according to the manufacturer's instructions. Exosomes were isolated from 3–4 independent experiments, with 11 individual readings for particle size determination per sample for each experiment.

### Mouse efficacy studies

All animal experiments were performed according to the guidelines and protocols approved by the Animal Care Committee at the University of Ottawa. Six week old Balb/C mice were obtained from Charles River Laboratories (Wilmington, MA, USA). Mice were injected with 1 × 10^5^ 4T1 cells resuspended in 100 μl PBS in the right hind flank. When tumors reached ~5 × 5 mm in size, mice were injected intratumorally with PBS or 1.9 × 10^9^ plaque forming units (pfu) AdFAST in 50 μl PBS. Tumor size was monitored three times a week using digital calipers. Mice were euthanized when the tumors reached 15x15 mm in size (endpoint). For tissue analyses, a second group of mice were injected when 4T1 tumors reached 7 × 7 to 10 × 10 mm in size and treated as described above. A third group of mice were injected with 1.4 × 10^8^ pfu AdEmpty. All mice used for histological analysis were sacrificed day 3 post vector injection and the tumor and liver were collected for further analysis. Samples were fixed with 10% formalin overnight and maintained in 70% ethanol. Samples were sectioned and stained with hematoxylin and eosin at the Morphology Unit of the Department of Pathology and Laboratory Medicine (University of Ottawa).

### Statistical analysis

SigmaStat 2.0 (San Jose, CA, USA) was used to assess statistical significance for all bar graphs using one-way ANOVA and Tukey's test. When the results did not follow a normal distribution, the Kruksal–Wallis one-way ANOVA on Ranks test was used. Dunn's test was applied instead of Tukey's test when required. To compare exosome yield, statistical analysis consisted of one-way ANOVA with *post-hoc* analysis by the Gabriel Pairwise Comparisons Test. For animal studies, the log-rank test was used to determine statistical significance of the Kaplan–Meier survival curve with Graphpad Prism 6.0 (La Jolla, CA, USA). All statistical tests were completed with *P*=0.05.

## Results

### Preliminary characterization of Ad infection of common mouse cancer cell lines

**Figure 1:**
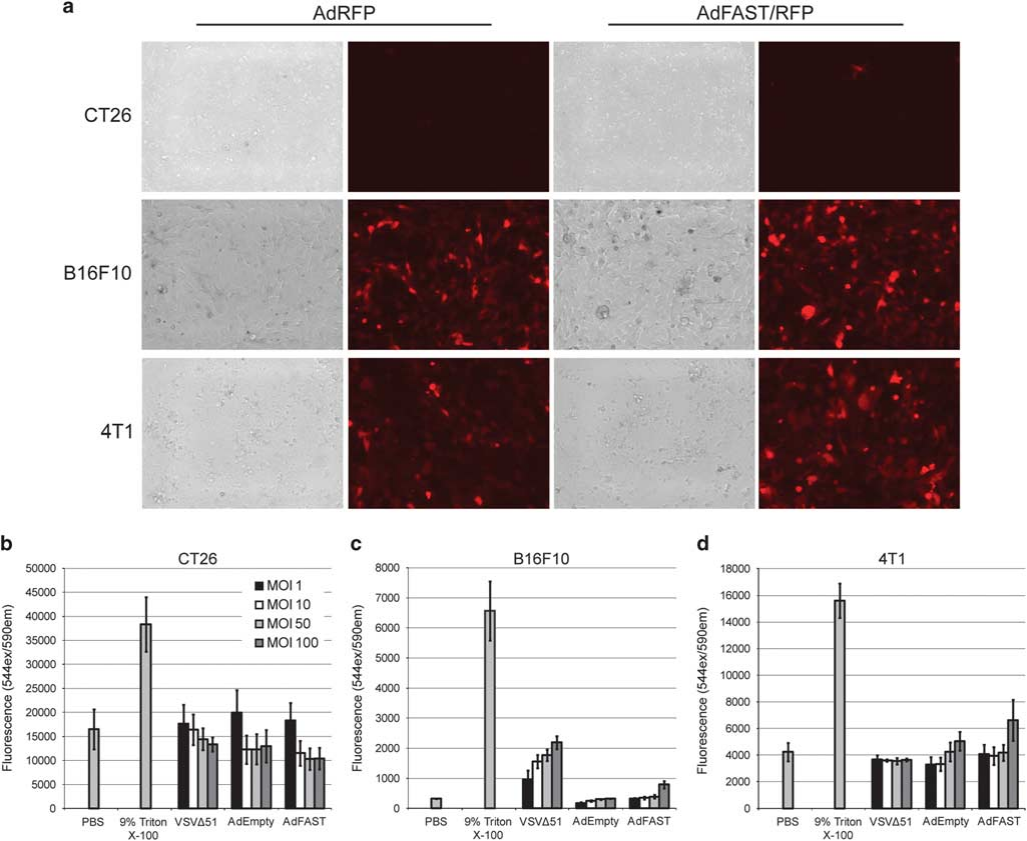
AdFAST/RFP infection efficiency in common mouse cancer cell lines *in vitro*. (**a**) CT26, B16F10 and 4T1 cells were infected with AdRFP or AdFAST/RFP at an MOI of 50 and visualized using fluorescence microscopy 48 hpi. (**b**) CT26 cells were infected with AdEmpty, AdFAST or VSVΔ51 at varying MOI from 1 to 100 for 72 h. Membrane permeability was assessed by determining the lactate dehydrogenase levels in the cell supernatant. Three independent experiments (*n*=3) were conducted in triplicate. The values graphed represent the average and the standard error of the mean. Experiments were repeated using the same protocol for (**c**) B16F10 and (**d**) 4T1 cells.

To examine whether AdFAST would provide a therapeutic benefit in an immunocompetent mouse model of cancer, we first examined several common mouse cancer cell lines for their susceptibility to Ad infection and FAST protein-mediated cell fusion. Mouse colon cancer CT26, melanoma B16F10 and mammary cancer 4T1 cells were infected with an E1 and E3-deleted Ad vector expressing RFP and FAST protein under the control of the CMV promoter/enhancer, and examined for gene expression and evidence of fusion 48 h later. At an MOI of 50, the CT26 cell line did not show evidence of infection, as no red cells were observed (Figure 1a). The 4T1 and B16F10 cell lines show a similar level of infection; however, neither cell line showed obvious signs of fusion at this MOI.

In the human lung adenocarcinoma A549 cell line, treatment with AdFAST caused a significant decrease in cell membrane integrity at as low an MOI as 1, whereas syncytium formation was only evident at a much higher MOI.^23^ Thus, we examined the relative membrane permeability of these three cell lines when infected with AdEmpty or AdFAST, by determining the quantity of lactate dehydrogenase (LDH) protein released into the supernatant at 72 hpi. CT26 and B16F10 cells showed no apparent difference in membrane integrity after infection at an MOI of up to 100 (Figures 1b and c). AdFAST infected 4T1 cells showed a modest increase in membrane permeability at MOI 100 compared with cells infected with AdEmpty (Figure 1d). Thus, we chose the 4T1 cell line for further characterization.

### 4T1 cells have low susceptibility to Ad infection

**Figure 2:**
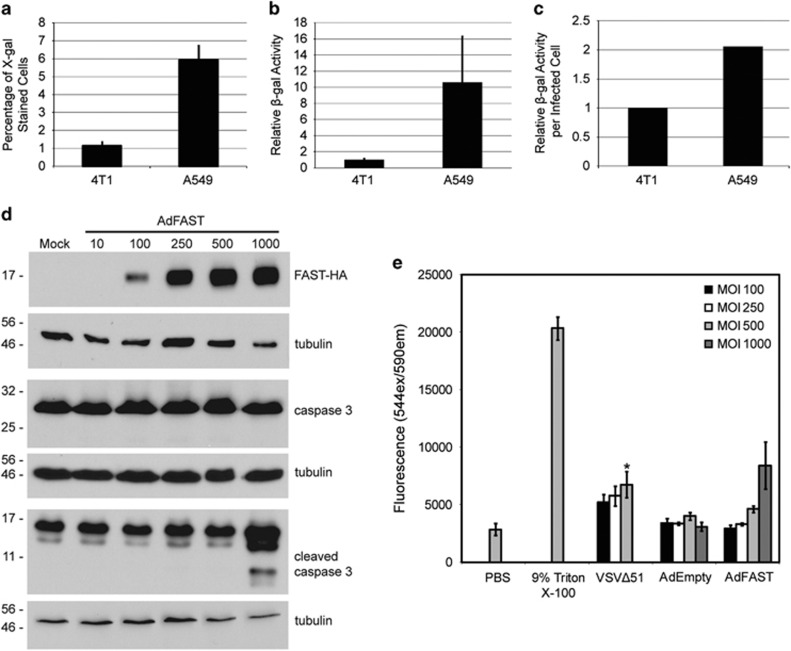
Ad shows lower infection efficiency and promoter activity in murine 4T1 cells relative to human A549 cells. 4T1 and A549 cells were infected with Adβ-Gal at an MOI of 1 for 24 h. Cells were either (**a**) fixed and stained with X-gal or (**b**) examined for β-galactosidase activity using a chemiluminescence assay. (**c**) The relative β-galactosidase activity per infected cell was determined (*n*=2). The average is shown for all experiments, and the error bars represent the standard deviation. (**d**) 4T1 cells were infected at varying MOI with AdFAST-HA, crude protein extracts prepared 72 hpi, and assayed for FAST protein expression, total caspase-3 and cleaved caspase-3 by immunoblot. Membranes were probed with antibody to tubulin to ensure equal protein loading. (**e**) 4T1 cells were infected with AdEmpty or AdFAST at MOI ranging from 100 to 1000, or VSVΔ51 at an MOI of 10 for 72 h. Membrane permeability was assessed by determining the lactate dehydrogenase levels in the cell supernatant. Three independent experiments (*n*=3) were conducted in triplicate. The values graphed represent the average and the standard error of the mean. *indicates significant difference between treatment and PBS (*P*<0.05).

Previously, we showed that under conditions in which AdFAST could replicate (that is, in E1-complementing 293 cells), rampant cell fusion was observed.^23^ However, under non-replicating conditions, high MOI of AdFAST was required to provide sufficient FAST protein expression to promote fusion, possibly due to a requirement for FAST protein to act as a multimer to mediate cell fusion.^34^ We therefore examined both the relative infection level and human CMV promoter activity in 4T1 versus human A549 cells, a cell line commonly used for Ad-based studies and the cell line that was used in our previous work.^23^ We infected 4T1 and A549 cells at an MOI of 1 with Adβ-Gal and determined the percentage of infected cells using X-gal staining and promoter activity using a chemiluminescent β-galactosidase activity assay 24 h later. Under these conditions, 1.2% of 4T1 cells stained blue, indicating infection by the Adβ-Gal virus, whereas A549 cells were infected at an efficiency of 5.9% (Figure 2a). Hence, Ad is able to infect and give rise to transgene expression in A549 cells approximately 5-fold more efficiently than 4T1 cells. An assessment of the promoter activity using the chemiluminescent assay showed that A549 cells had a 10.6-fold increase in reporter gene activity compared with 4T1 cells at this MOI (Figure 2b). When β-gal activity is normalized to the number of infected cells, A549 cells had an ~2.1-fold higher level of β-gal expression per infected cell compared with 4T1 cells (Figure 2c). Therefore, although the level of protein expression driven from the CMV promoter in an Ad vector is not dramatically different between the two cell lines, a higher MOI is likely required to achieve the same degree of cell infection, and thus overall level of FAST protein expression, in 4T1 cells compared with A549 cells.

We showed previously that Ad-mediated expression of FAST in A549 cells induced significant apoptosis at an MOI as low as 10.^23^ As 4T1 cells showed an ~10-fold reduction in ability to support Ad transgene expression compared with A549 cells, we examined whether higher MOI infection with AdFAST could induce apoptosis in the 4T1 cell line. 4T1 cells were infected with varying MOI (10–1000 pfu per cell) of AdFAST-HA, and crude protein samples were analysed 72 h later for expression of FAST and cleavage of caspase-3, a marker of apoptosis. As shown in Figure 2d, although FAST expression could be detected in cells at an MOI of 100, apoptosis was only detected at very high MOI. Similarly, using the LDH release assay to evaluate cell membrane integrity, cells infected with AdFAST at an MOI of 1000 showed an almost threefold increase in membrane permeability compared with AdEmpty infected cells at the same MOI, but this effect did not reach statistical significance (Figure 2e). Taken together, these results indicate that the relatively poor ability of Ad to infect 4T1 cells necessitates the use of high MOI to achieve the levels of FAST protein necessary to induce cell death.

### FAST protein expression in 4T1 cells causes cell fusion, decreases cellular metabolic activity and induces apoptosis

**Figure 3:**
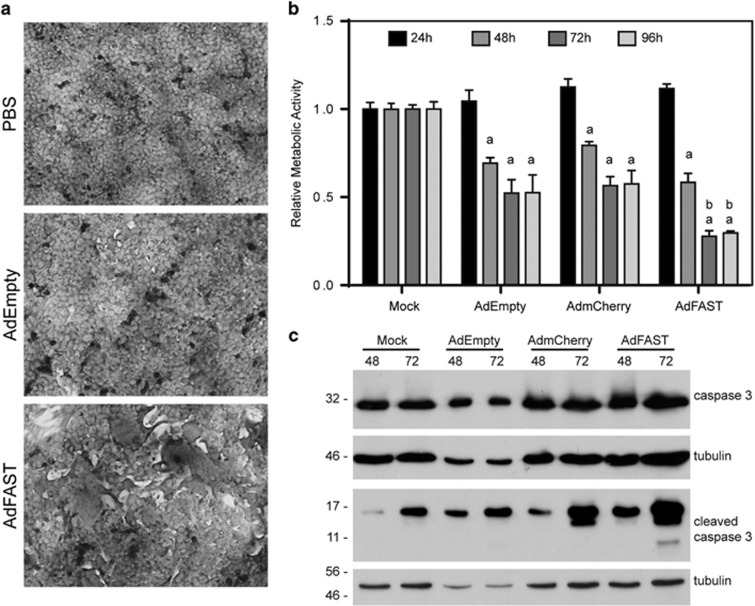
Infection of 4T1 cells with AdFAST promotes cell fusion, reduces cellular metabolic activity, and enhances apoptosis. (**a**) 4T1 cells were infected with AdEmpty or AdFAST at an MOI of 1000, or mock infected with PBS. Cells were fixed and stained with Giemsa stain 48 hpi. Images depicted are representative of two replicates (*n*=2) (20 × objective). (**b**) 4T1 cells were infected with AdEmpty, AdmCherry or AdFAST at an MOI of 1000, or mock infected with PBS, and relative metabolic activity was determined using an MTS assay over a time course of 96 h. Results depict the average of three independent experiments (*n*=3) performed in triplicate. Error bars show the standard error of mean. ^a^*P*<0.001 compared with 24 h within a treatment group. ^b^*P*<0.001 for AdFAST compared with AdEmpty- and AdmCherry-treated cells. (**c**) 4T1 cells were infected at varying MOI with AdFAST-HA, crude protein extracts prepared 72 hpi, and assayed for total caspase-3 and cleaved caspase-3 by immunoblot. Membranes were probed with antibody to tubulin to ensure equal protein loading.

We further examined the effect of FAST protein expression on 4T1 cell function by examining cell fusion and metabolic activity of AdFAST-treated cells compared with control vector. First, 4T1 cells were infected with AdEmpty or AdFAST at an MOI of 1000, and stained with Geimsa stain to aid in visualizing fused cells. As shown in Figure 3a, neither PBS nor AdEmpty-treated cells showed any evidence of cell fusion. In contrast, 4T1 cells treated with AdFAST showed altered morphology with several regions of cell fusion evident per field of view. Thus, FAST protein expression from an Ad vector can cause 4T1 cell fusion.

We next examined whether treatment of 4T1 cells with AdFAST altered cellular metabolic activity relative to control vector-treated cells. Treatment of 4T1 cells with an MOI of 1000 with AdEmpty or AdmCherry resulted in a 30–40% decrease in metabolic activity at time points beyond 24 hpi (Figure 3b). However, AdFAST caused a more pronounced decrease in metabolic activity of the cells, particularly at later time points, where an almost 80% decrease in metabolic activity was observed. Consistent with this, although we observed evidence of apoptosis in all treatment groups, AdFAST-treated cells had higher levels of the cleaved caspase-3 (Figure 3c), suggesting that AdFAST caused greater cell death.

### Ad-mediated expression of FAST protein promotes exosome release from 4T1 cells

**Figure 4:**
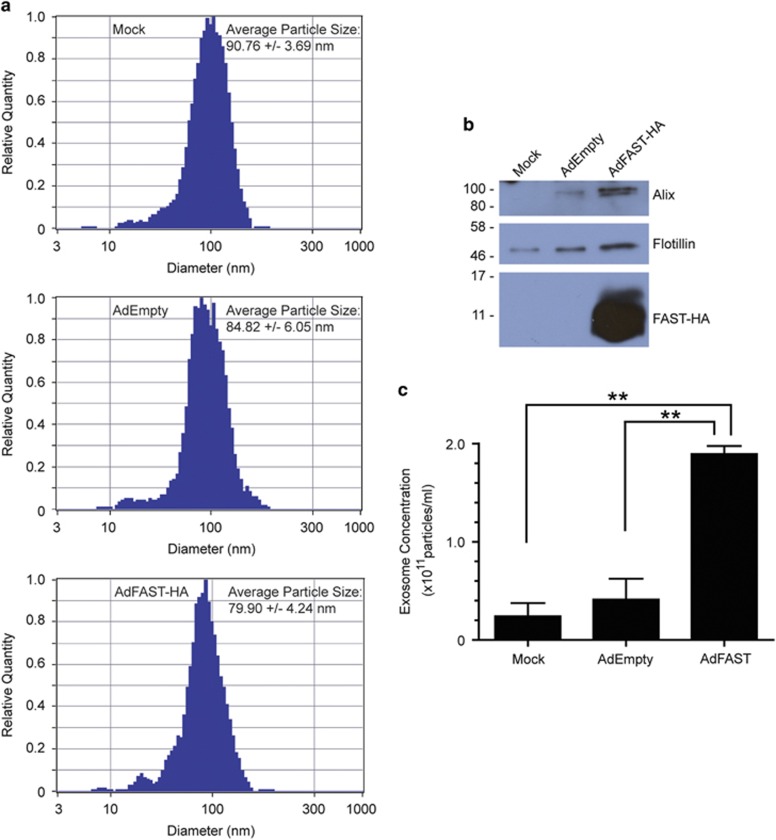
Treatment of 4T1 cells with AdFAST enhances release of exosomes. (**a**) 4T1 were infected with AdEmpty or AdFAST-HA at an MOI of 1000, or mock infected with PBS and exosomes were isolated from the medium 72 hpi using Exoquick. An aliquot of the resulting purified exosomes were analyzed by Zetaview (Particlemetrix, Germany) for particle size. Shown is one representative histogram of size distribution for each treatment along with the average +/− standard deviation of the particle size for 3–4 independent experiments. (**b**) 4T1 were infected with AdEmpty or AdFAST-HA at an MOI of 1000, or mock infected with PBS, and exosomes were isolated from the medium 72 hpi. Equal volume of the resulting exosome samples were separated by SDS–PAGE, and analysed by immunoblot for exosome markers Alix and Flotillin, and for the HA tag on FAST protein. (**c**) The concentration of exosome particles isolated from media was quantified using Zetaview according to the manufacturer's instructions. Exosomes were isolated from 3–4 independent experiments. Bars represent mean±standard deviation. ** indicates significant differences between groups (*P*<0.0001).

Previous studies showed that expression of fusogenic proteins in cancer cells can lead to the enhanced release of exosome-like molecules called syncytiosomes.^16^ Importantly, these syncytiosomes contain tumor antigens and are effective at priming antigen presenting cells to elicite the formation of cancer-specific T cell that can eliminate the tumor and provide long lasting anti-tumor immunity.^16, 17, 18^ To determine if Ad-mediated expression of FAST protein also results in the enhanced release of exosomes, we infected 4T1 cells with AdEmpty or AdFAST-HA at an MOI of 1000, and isolated exosomes from the medium 72 hpi using a commercially available kit. An analysis of particle size showed that these were indeed within the size range of exosomes (Figure 4a). Equal volumes of the resulting exosome samples were analyzed by immunoblot for the expression of two protein markers of exosomes, Alix and Flotillin. As shown in Figure 4b, protein samples from AdFAST-treated cells showed a higher level of both Alix and Flotillin suggesting enhanced quantities of exosomes were in the medium relative to mock- or AdEmpty-treated cells. Consistent with this observation, direct quantification of the number of exosomes in the isolated samples showed that there was an ~fivefold increase in exosomes released from AdFAST-treated cells compared with AdEmpty-treated cells (Figure 4c). Therefore, Ad-mediated expression of FAST protein in 4T1 cells results in enhanced release of exosomes, which may be bioavailable to enhance immune responses to tumor-associated antigens thereby possibly contributing to therapeutic efficacy.

### Ad-mediated FAST protein expression does not reduce tumor burden or promote survival in subcutaneous 4T1 tumors in immunocompetent mice

**Figure 5:**
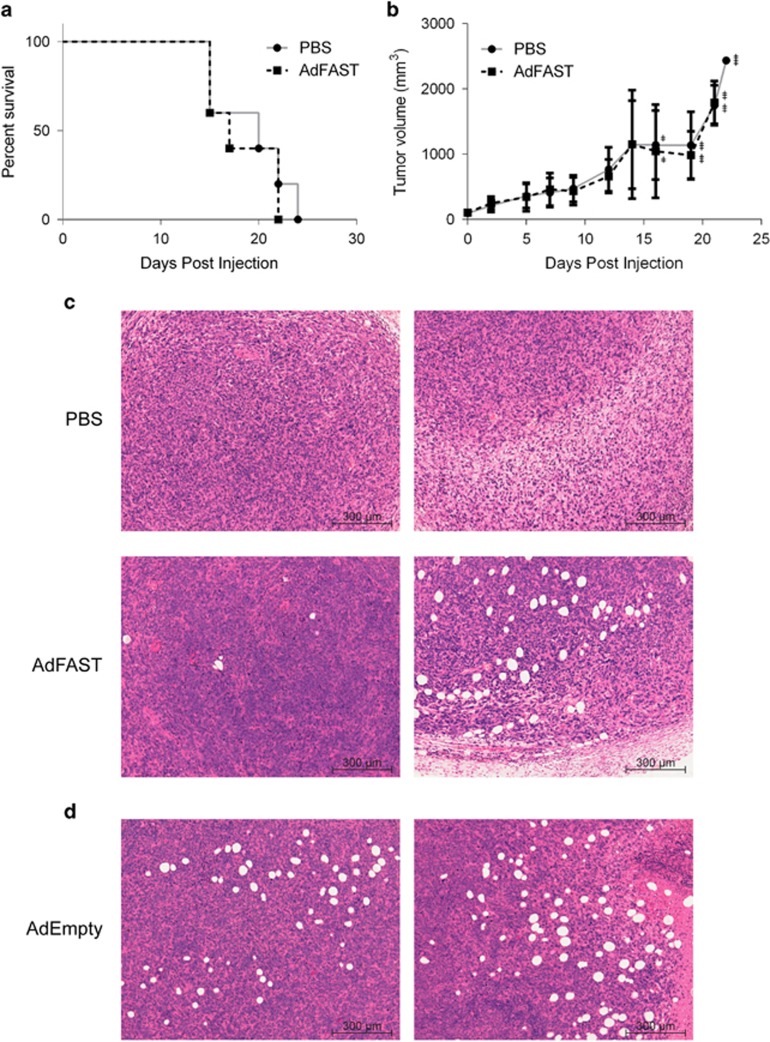
AdFAST does not promote survival or reduce tumor growth in immunocompetent Balb/C mice with subcutaneous 4T1 tumors. (**a**) Kaplan–Meier survival curve for 4T1 tumor-bearing mice treated with AdFAST. Balb/C mice with subcutaneous 4T1 tumors were intratumorally injected with PBS or 1.9 × 10^9^ pfu AdFAST. Each treatment group consisted of five mice. (**b**) The average size of tumors treated with PBS or AdFAST as described in (**a**). The error bars represent the standard deviation. The + symbols show the number of mice that had reached endpoint by the time measurements were taken. (**c**) Subcutaneous 4T1 tumors from Balb/C mice were removed, fixed, sectioned and stained with hematoxylin and eosin stain 3 days post injection. Images are representative from a group of three mice per treatment. (**d**) Balb/C mice with subcutaneous 4T1 tumors were intratumorally injected with 1.4 × 10^8^ pfu AdEmpty. Three days post injection, tumors were excised, fixed, sectioned and subjected to hematoxylin and eosin stain.

We next examined whether AdFAST could provide a therapeutic effect in an immunocompetent 4T1 tumor model *in vivo*. Balb/C mice with 4T1 subcutaneous tumors were injected with PBS or 1.9 × 10^9^ pfu AdFAST and tumor volume and survival were monitored at varying time points post-treatment. Treatment of tumor-bearing mice with AdFAST did not alter the rate of tumor growth nor did it enhance survival (Figures 5a and b). Tumors that were excised 3 days post injection showed no obvious signs of cell fusion (Figure 5c). However, in AdFAST-treated tumors, we observed numerous lesions located near tumor margins, which appeared as holes in the tissue. As we were unsure whether this was due to FAST protein expression or the Ad vector itself, we injected 4T1 tumor-bearing mice intratumorally with 1.4x10^8^ pfu AdEmpty and removed the tumors 3 days post injection for analyses. AdEmpty-treated tumors also had similar lesions, indicating the effects seen were due to the Ad vector and not expression of FAST (Figure 5d). As Ad normally accumulates in the liver following both systemic delivery and local, intratumoral injection,^35, 36^ livers were also collected at the time of sacrifice. There were no obvious differences in pathology of livers from PBS or AdFAST-treated mice (data not shown). Therefore, although AdFAST can cause fusion and alter metabolic activity of 4T1 cells *in vitro*, in the 4T1 immunocompetent mouse tumor model AdFAST showed no obvious therapeutic benefit *in vivo*.

## Discussion

Our previous work had examined the effects of FAST protein expression in the A549 lung adenocarcinoma model *in vitro* and in an A549 xenograft nude mouse model *in vivo.*^23^ Although we observed evidence of fusion and enhanced cell death *in vitro*, treatment of established tumors in the immunocompromised mice with AdFAST did not provide a therapeutic benefit. Previous studies have shown that part of the efficacy of fusogenic proteins can be due to enhanced release of tumor-associated antigen-containing exosome-like particles, referred to as syncytiosomes, from the fused cell, which can subsequently act to stimulate immune-mediated rejection of the tumor.^16^ This effect would be absent in our A549 xenograft studies. We therefore investigated whether AdFAST would have a stronger therapeutic effect in an immunocompetent mouse tumor model.

Of the three mouse cancer cell lines we investigated, we found that Ad infected B16F10 and 4T1 tumor cells with a similar efficiency (Figure 1a). However, infection of these two cell lines with AdFAST showed that the 4T1 cells had a higher degree of FAST-induced cell membrane permeability (Figures 1 and 2), although a very high MOI was required for this effect. 4T1 cells showed a lower overall efficiency of infection relative to A549 cells (~5-fold lower, Figure 2a) and also approximately half the relative amount of expression per infected cell (Figure 2c). Within AdFAST, FAST expression is regulated by the human CMV immediate early enhancer/promoter. Previous studies have shown that the CMV enhancer/promoter is less active in murine cells than in human cells,^37^ consistent with our observations. Thus, although the 4T1 model may be adequate to evaluate the efficacy of AdFAST in an immunocompetent mouse model, we would predict that higher amounts of virus may be required to see an effect.

Although we achieved significant levels of FAST protein expression in 4T1 cells, especially at high MOI (Figure 2d), we observed only modest levels of cell fusion (Figure 3a). It is possible that FAST protein expression caused formation of small syncytia at lower MOIs, which were not readily detectable in our fusion assay. However, there may be fundamental differences between human and mouse cells that confer differences in ability to fuse. Previous work with GALV and MV fusogenic proteins showed limited fusion activity in mouse models,^16^ which suggests that this species may lack cellular factors necessary for fusion. Regardless, the low-level of fusion we observed in 4T1 cells was accompanied by a significant decrease in metabolic activity (Figure 3b), which appeared to progress to cell death due to caspase-3-mediated apoptosis (Figures 2d and 3c). It is possible that a significant amount of FAST protein may mislocalize to the membrane of cellular organelles, such as the mitochondria, leading to metabolic failure and ultimately cell death, as has been observed for other fusogenic proteins.^38^ Cell death through this mechanism would be independent of overt cell fusion. Nevertheless, these results suggested that AdFAST warranted testing for efficacy *in vivo.*

Treatment of 4T1 tumor-bearing mice with AdFAST did not result in enhanced survival (Figure 5a) or a reduction in tumor growth (Figure 5b). Previous studies with replication-defective Ad vectors expressing RSV or MV fusion proteins showed that although these vectors mediated limited fusion in mouse cells *in vitro*, they were still able to act as effective sole therapeutics to reduce tumor burden in syngeneic mouse models.^9, 12^ A similar effect was not observed for our experiments with the FAST protein. Work by Bateman *et al.*^16^ demonstrated that fused syncytia have enhanced release of exosome-like particles. These syncytiosomes could load tumor-associated antigens into DC, and were more effective at stimulating anti-tumor immunity than cells dying by other means. We had hoped that a similar mechanism would occur for FAST-expressing tumor cells. Although we showed that AdFAST-treated 4T1 cells have an enhanced release of exosomes *in vitro* (Figure 4), this did not translate to a greater therapeutic effect *in vivo*. However, the 4T1 model is characterized as having a relatively low inherent immunogenicity,^39^ and any FAST-mediated indirect effects on anti-tumor immunity may be ineffective at surmounting this obstacle.

We did not observe evidence of cell fusion in tumor sections from AdFAST-treated mice (Figure 5c). However, we did observe a large number of pockets or holes in tumor sections from animals receiving treatment with either AdEmpty or AdFAST, but not in animals injected with PBS, suggesting these structures were due to administration of the Ad vector and independent of transgene expression. Interestingly, we also observed a significant reduction in metabolic activity when 4T1 cells were treated with our two control vectors AdEmpty and AdmCherry *in vitro* (Figure 3b). Although these vectors are deleted of the E1 (and E3) region, and are thus largely replication-defective, they still have the ability to express some viral proteins, such as those encoded by E4.^40^ One of the E4 open reading frames, E4ORF4, is capable of inducing p53-independent cell death through a non-classical apoptotic mechanism.^41^ It is possible that low-level expression of some of these viral proteins contributes to the formation of these structures, but the mechanism is unclear. Regardless, we did not observe a difference in rate of tumor growth between Ad- and PBS-treated animals, indicating that the presence of these structures was not associated with tumor stasis nor tumor regression.

Our study shows that Ad-mediated p14 FAST protein expression did not induce extensive cell–cell fusion and had modest effects on membrane permeability and cellular metabolic activity in the mouse cancer cell lines examined. Moreover, AdFAST did not have an enhanced therapeutic benefit in the immunocompetent 4T1 mouse mammary cancer model compared with mock-treated mice. Our results suggest that a modified treatment regime, vector modifications or additional immunostimulatory transgenes may be required to induce a therapeutic effect with AdFAST in immunocompetent *in vivo* models of cancer.
